# Epigenetic associations with adolescent grey matter maturation and cognitive development

**DOI:** 10.3389/fgene.2023.1222619

**Published:** 2023-07-17

**Authors:** Dawn Jensen, Jiayu Chen, Jessica A. Turner, Julia M. Stephen, Yu-Ping Wang, Tony W. Wilson, Vince D. Calhoun, Jingyu Liu

**Affiliations:** ^1^ Tri-Institutional Center for Translational Research in Neuroimaging and Data Science (TReNDS), Georgia State University, Georgia Institute of Technology, Emory University, Atlanta, GA, United States; ^2^ Neuroscience Institute, Georgia State University, Atlanta, GA, United States; ^3^ Department of Computer Science, Georgia State University, Atlanta, GA, United States; ^4^ Wexnar Medical Center, Department of Psychiatry and Behavioral Health, Ohio State University, Columbus, OH, United States; ^5^ The Mind Research Network, Albuquerque, NM, United States; ^6^ Department of Biomedical Engineering, Tulane University, New Orleans, LA, United States; ^7^ Institute for Human Neuroscience, Boys Town National Research Hospital, Omaha, NE, United States; ^8^ Psychology Department and Neuroscience Institute, Georgia State University, Atlanta, GA, United States

**Keywords:** adolescent development, grey matter, methylation, neuroimaging epigenetics, cognition, sMRI

## Abstract

**Introduction:** Adolescence, a critical phase of human neurodevelopment, is marked by a tremendous reorganization of the brain and accompanied by improved cognitive performance. This development is driven in part by gene expression, which in turn is partly regulated by DNA methylation (DNAm).

**Methods:** We collected brain imaging, cognitive assessments, and DNAm in a longitudinal cohort of approximately 200 typically developing participants, aged 9–14. This data, from three time points roughly 1 year apart, was used to explore the relationships between seven cytosine–phosphate–guanine (CpG) sites in genes highly expressed in brain tissues (*GRIN2D*, *GABRB3*, *KCNC1*, *SLC12A9*, *CHD5*, *STXBP5*, and *NFASC*), seven networks of grey matter (GM) volume change, and scores from seven cognitive tests.

**Results:** The demethylation of the CpGs as well as the rates of change in DNAm were significantly related to improvements in total, crystalized, and fluid cognition scores, executive function, episodic memory, and processing speed, as well as several networks of GM volume increases and decreases that highlight typical patterns of brain maturation.

**Discussion:** Our study provides a first look at the DNAm of genes involved in myelination, excitatory and inhibitory receptors, and connectivity, how they are related to the large-scale changes occurring in the brain structure as well as cognition during adolescence.

## Introduction

Epigenetic regulation of gene expression occurs through a variety of mechanisms that include modification of histone, DNA methylation, and noncoding RNA regulation. These mechanisms modulate the accessibility of chromatin to transcriptional machinery, leading to altered expression of genes (Moore et al., 2013; Perri et al., 2017; Mangiavacchi et al., 2023). Methylation of the DNA, the more well-studied of these epigenetic processes, occurs when a methyl group attaches to a cytosine pyrimidine (CpG) ring, causing either an increase or decrease in gene expression, as well as alternative splicing during transcription of genes (Dupont et al., 2009). This intricate orchestration of gene expression and regulation is extremely plastic and sensitive to developmental cues (Dupont et al., 2009). While there has been extensive research demonstrating the strong impact methylation (DNAm), in particular, has on fetal brain development in humans (Schneider et al., 2016), the invasive nature of harvesting brain tissue for analysis has precluded the study of its role in neurodevelopment during human adolescence (Wheater et al., 2020). Recent developments have made it possible to use peripheral tissue samples such as saliva to assess DNAm changes non-invasively (Walton et al., 2016; Lin et al., 2018; Proskovec et al., 2020), affording researchers the opportunity to explore these molecular underpinnings of brain development beyond the post-natal stage.

Animal studies have shown that during adolescence, a period of neural maturation, there are large scale epigenomic changes occurring (Mychasiuk and Metz, 2016). While studies of normal development are few, a cross-sectional study in adolescent rats demonstrated that epigenetic regulators of specific genes, such as stress responders (*Hsp10*), cellular regulators, (*Sirt1*), growth factors (brain-derived neurotrophic factor), and glial-specific genes (*Gfap*), were expressed differentially, with greater overall expression in females than males and increased levels of expression of these regulators found in the prefrontal cortex compared to the hippocampus (Mychasiuk et al., 2011). A longitudinal study in rats showed adolescence-related reductions in the expression of dopamine receptors in cortical output neurons ranging from the prefrontal cortex to the nucleus accumbens (Shaw et al., 2006).

Recent research has shown that DNA methylation biomarkers gathered from peripheral tissue samples like blood and saliva have relationships with aspects of structural and functional measures of the brain (Walton et al., 2016; Lin et al., 2018; Proskovec et al., 2020). One study collected resected brain tissue in conjunction with saliva, blood, and buccal samples from 27 subjects undergoing neurosurgery for intractable epilepsy to calculate within and between subject correlations of DNA methylation of CpGs between peripheral tissue and brain. This study found that the correlation between saliva and brain epigenome-wide profiles is as high as r = 0.90 and 15.1% of individual CpGs in saliva correlated to brain at a nominally significant level (Braun et al., 2019).

Since the use of DNAm biomarkers gathered from peripheral tissue have made studying epigenetic mechanisms without harvesting brain tissue possible, there have been a few longitudinal studies looking at the changes occurring during human adolescence (Sanders et al., 2022). A study published in 2019 investigated the pre- and post-adolescent changes in DNAm, finding that between the ages of 10–18 years of age, roughly 15 k CpGs showed significant changes in DNAm (Han et al., 2019). Another study published in 2021 found that DNAm mediated the relationship between childhood adversity and the symptoms of depression across adolescence (Smith et al., 2021). Yet, there is little existing research regarding epigenetic influences on normal cognitive and brain development during adolescence (Wheater et al., 2020).

The neural development that occurs during human adolescence has been well-researched. Repeated longitudinal structural magnetic resonance imaging (sMRI) studies, where grey matter (GM) density and volume are measured as an indirect reflection of neuronal, dendritic and synaptic processes, glia and vasculature in the brain, consistently demonstrate that typical human brain development involves a birth-to-adolescence increase in GM volume that is then followed by a decrease during adolescence (10–25 years of age) that stabilizes in early adulthood (25 years of age and up) (Gogtay et al., 2004; Krongold et al., 2017; Tamnes et al., 2017). This reduction in GM volume that is seen throughout adolescence is widespread in the brain, but with regional variability as age increases. In general, lower-order regions are first to mature and then the higher-order association areas follow (Gogtay et al., 2004). This reorganization of the structure of the brain is accompanied by behavioral changes that manifest as overall improvement across a broad spectrum of cognitive measures that include improved attention, increased inhibition and control, improved memory and metacognition, continued development of cognitive self-regulation, increased speed of processing capacity, as well as more nuanced calibration of risk and reward (Steinberg, 2005).

Our study will take advantage of the recent advances in DNAm analysis to model the interactions between DNAm, GM volume changes, and cognitive development during human adolescence. To do this, we have used the Developmental Chronnecto-Genomics (Dev-CoG): A Next-Generation Framework for Quantifying Brain Dynamics and Related Genetic Factors in Childhood, a longitudinal cohort of roughly 200 typically developing subjects aged 9–14. This project collected brain imaging, cognitive assessments, DNA genetics and methylation data over three time points, with roughly 1 year between each (Stephen et al., 2021). With this data, we will identify CpGs sites where DNAm changed significantly over that time, and assess the relationship of DNAm change with the GM volume and cognitive performance changes. We will also investigate the relationships between the rates of changes between DNAm and the rates of GM volume change and cognitive performance respectively. Based on the current understanding of neural development and cognitive improvement during adolescence, we expect to see changes in DNAm in genes that play a role in GM volume changes and improved cognitive function over time.

## Methods

### Cohort

All data were collected as part of the Dev-CoG study (Stephen et al., 2021) and shared through an agreement with the project leaders. Subjects were recruited by the Mind Research Network (MRN) and the University of Nebraska Medical Center (UNMC). Approval from the relevant institutional review board at each data collection site (Advarrra IRB—MRN and UNMC IRB—Nebraska) and data sharing across study sites was written into the consent forms and the study protocols (Stephen et al., 2021). Images, saliva samples, and cognitive tests were collected from 200 male and female subjects between the ages of 9–14 over three time points, roughly a year between each collection. The inclusion criteria for the study were: English speaking, age 9–14 years at enrollment and both child and parent were able and willing to assent/consent to the study. The exclusion criteria for the study were: current pregnancy, unable to consent/assent, history of developmental delays or disorders (or an individual education plan indicative of a developmental delay/disorder), history of epilepsy or other neurological disorders, parental history of major psychiatric or neurological disorders, self-reported prenatal exposure to alcohol or drugs, medication use, contraindication to MRI (MRI screening form was reviewed), or metal orthodontia (e.g., braces or spacers) (Stephen et al., 2021). The mean age at enrollment was 11.3 years old (full range 9–14) for the entire group. In the repeated measure analysis reported below, 106 subjects were analyzed (48 girls, 58 boys), who had a mean baseline age of 11.75 years old. The multivariate analysis of difference maps included 138 subjects for deltaT1 (mean baseline age 11.83 years old, 67 females, 71 males) and 81 subjects for deltaT2 (35 females, 46 males). The sample size differences between the repeated measures and multivariate analyses are due to the flexibility of the linear mixed-effects model when dealing with missing data (Gabrio et al., 2022). See Table 1 for more demographic information.

**TABLE 1:** General Demographic information: MRN - Mind Research Network, UNMC - University of Nebraska Medical College, BIPOC - Black, Indigenous, and People Of Color, WASI II IQ - Wechsler Abbreviated Scale of Intelligence, a general IQ test, a score between 90–102 is considered average, SES - Socioeconomic Score.

Demographics	MRN (101)	UNMC (102)
Mean age at enrollment (range)	11.3 (9–14)	11.2 (9–14)
Gender (M/F)	51M/50F	51M/51F
Race (Caucasian/BIPOC)	86/15	87/15
Ethnicity (% Hispanic)	41.6%	7.8%
Mean WASI-II IQ (Range)	108.6 (72–139)	112.1 (68–148)
Mean SES (Range)	42.6 (17–66)	48.2 (15–65)

### DNAm preprocessing

Saliva DNAm was assessed for each subject using the Illumina HumanMethylation850 (850 k) microarray, which measures CpG methylation across ∼850,000 probes covering 99% of gene promoters. Standardized quality control procedures and quantile normalization was performed using the minfi Bioconductor package in R (version 3.6.2) (Aryee et al., 2014). Red and green channel intensities were mapped to the methylated and unmethylated status, with average intensities used to check for low quality samples. Beta values reflect the degree of methylation, (from 0–1) and were calculated for each CpG, for each subject. Principal component analyses (PCA) were performed on the beta values to identify any samples more than three standard deviations away from the median on any of the first four components. These were considered outliers and removed. Samples where the genetically determined sex differed from the self-reported value were also removed. 20 duplicate DNA samples were included in each batch to ensure measurement reliability. Samples processed in different batches were merged at this stage. Stratified quantile normalization was then applied across sample, using the minfi PreprocessQuantile function. The cell proportions for each DNAm sample were calculated by implementing the estimateCellCounts function in minfi, using a combined reference of five types of blood cells (B cells, CD8T and CD4T cells, NK-LGL cells, monocytes, and granulocytes), as well as epithelial cells (GSE46573) (Aryee et al., 2014). In this cohort, the proportion of B cells, CD8T, CD4T, monocytes, and NK-LGL cells were zero. The estimated proportion of granulocytes and epithelial cells was 70 percent and 30 percent respectively. The cell type effect was regressed out from all the samples to account for the change of cell proportion over time. Batch effects were corrected by using the R package Combat, which assumes normalized data and equalizes the mean from all batches, making negative values possible (Johnson et al., 2007).

### CpG selection

**FIGURE 1:**
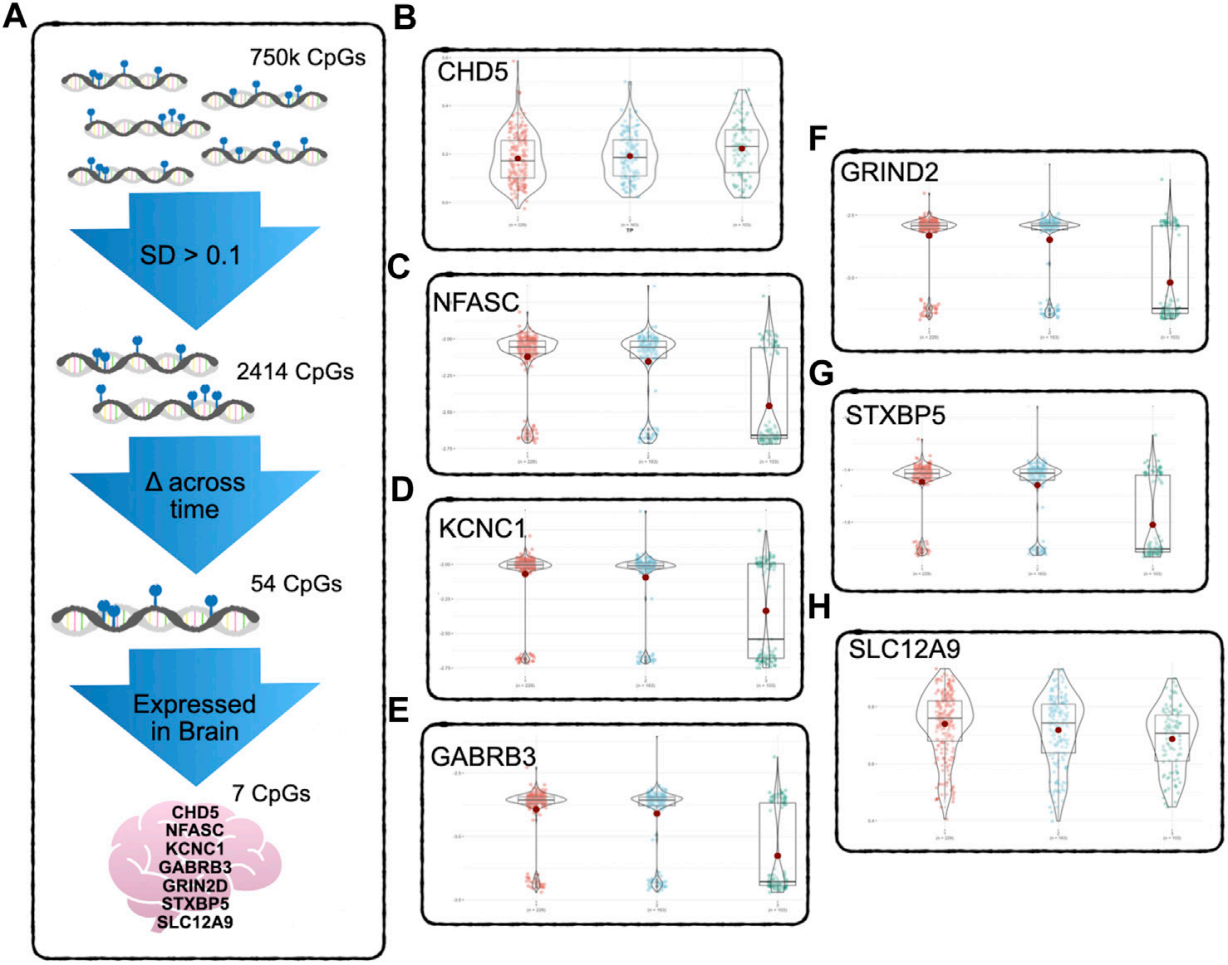
DNAm Filtering Process and Results - **(A)** Starting with 750k CpGs after preprocessing and QC, only CpGs above the 0.1 standard deviation of variability were kept, reducing the number of CpGs to 2414. A pairwise *t*-test was used to detect CpGs with significant change across time points (FDR corrected for multiple testing). Only 54 CpGs showed significant change across all three timepoints. Of these, seven were found to be regulating genes that according to the Human Protein Atlas are expressed in the brain. **(B–H)** show the significant change across all three timepoints. The CpG regulating *CHD5* **(B)** was the only one of the seven to show significant increase in methylation, the other six CpGs decreased in methylation over time. See Table 2 for detailed results.

At this stage, there were approximately 750K CpG sites retained per subject, per time point. We kept only CpG sites with a sample standard deviation of 0.1 or higher at the first time point, to ensure that baseline variability across subjects exceeded measurement variability (Duan et al., 2021). This reduced the number of CpG sites to 2414. Since we were primarily interested in CpG sites whose DNAm values were changing over the three time points of data collected, the CpG sites were further filtered by keeping the sites that had changed significantly in methylation between time points, using paired t-tests. After FDR correction for multiple testing (Benjamini and Hochberg, 2000), 54 CpG sites during deltaT1 (time point 2 - time point 1) and 465 CpGs during deltaT2 (time point 3 - time points 2) were identified as changing significantly, and 54 CpGs were in common. The Infinium MethylationEPIC Manifest file (Hansen, 2016) was used to identify the annotated genes of these 54 CpGs. Cross-referencing these genes with the Human Protein Atlas (Uhlén et al., 2015), seven of these CpGs were found to be located on genes highly expressed in the brain. Refer to Figure 1A for a diagram of the filtering process. The subsequent analyses focused on these seven CpG sites. See Table 2 for detailed results. A *post hoc* gene set enrichment analysis (GSEA) was performed using the GENE2FUNC function in FUMA (https://fuma.ctglab.nl/) (Watanabe et al., 2017). Correction for multiple testing was done using the Benjamini–Hochberg method. To further confirm tissue specificity, genes regulated by these seven CpGs were also tested for enrichment in specific human tissues FUMA. See Supplementary Table S2 for complete results.

**TABLE 2:** Identification of the seven CpGs and change in DNAm over time: These CpGs regulate genes identified by the Infinium MethylationEPIC Manifest file, cross-referenced by the Human Protein Atlas, and verified through FUMA tissue expression analysis as being highly expressed in the brain. TP1-TP2, n = 163, TP2-TP3, n = 103. All *p*-values are FDR-corrected for multiple tests.

CpGs	Gene	Location	Regulatory Activity	T1-T2 (t/p)	T2-T3 (t/p)
cg01008256	SLC12A9	Exon	Highly Active	−4.87/0.003	−4.31/5.3E-4
cg23841819	NFASC	Exon	Active	−4.14/0.004	−12.38/5.2E-20
cg01483824	GRIN2D	Exon	Highly Active	−4.31/0.003	−13.05/7.0E-21
cg15205435	CHD5	Intron	Highly Active	3.57/0.021	4.69/1.50E-4
cg15866800	STXBP5	Intron	Highly Active	−4.36/0.003	−13.21/6.2E-21
cg14859324	GABRB3	Intron	Highly Active	−4.30/0.003	−9.35/1.3E-13
cg26703758	KCNC1	Intron	Active	−4.36/0.003	−13.44/6.0E-21

### sMRI data

T1-weighted structural MRI images were collected at the MRN site on a Siemens 3T TrioTim scanner, and at UNMC site on a Siemens 3T Magnetom Skyra and Prisma scanners, all with a 32-channel radio frequency coil. Scanning parameters were equilibrated as much as possible. The sMRI images were reoriented and registered to a cohort specific template, created using the ANTS multivariate template generator, and resampled to 2 mm × 2 mm × 2 mm (Andersson et al., 2007a; Andersson et al., 2007b; Avants et al., 2008; Sanchez et al., 2012). Using FAST in FSL, a high-dimensional normalization pipeline, the non-brain tissues was removed, and the grey matter, white matter, and cerebral spinal fluid segmented, leaving normalized, modulated, Jacobian-scaled grey matter images (Zhang et al., 2001) that were smoothed by a 4 mm × 4 mm x 4 mm full width at half maximum Gaussian kernel (Smith and Brady, 1997). The resultant grey matter images then had scanner differences regressed out using a simple linear regression with age and sex as covariates. Two subjects were removed due to movement (framewise displacement from rs-fMRI) above 3 standard deviations from the mean of the group.

To calculate the rate and direction of change across time points, grey matter volumes from TP1 were subtracted from TP2 to create the deltaT1 difference map for each individual, and TP2 was subtracted from TP3 to create the deltaT2 difference map. An independent component analysis (ICA) built in the GIFT toolbox (SBM v1.0b) (Xu et al., 2009) was then applied to the difference maps to extract components/brain networks, where distributed brain regions showed covarying patterns of GM volume changes. The components’ associated loadings reflect these brain regions’ variation across subjects. Using the minimum description length (MDL) criterion (Calhoun and Adali, 2009), seven components were extracted from the GM volume changes of deltaT1, identifying our brain networks of interest for this study. The direction of the ICA loadings were confirmed through a voxel-based morphometry (VBM) analysis in FSL (Smith et al., 2004), where positive loadings indicate increases in GM volume and negative loadings indicate decreases in GM volume. The spatial maps of these seven components were projected onto the subjects’ deltaT2 GM images, as well as to the subjects’ GM images at each time point to ensure uniformity of comparison.

### Cognitive data

The age-uncorrected standard scores from the following NIH cognitive toolbox tests (Denboer et al., 2014) were collected from each subject: the Picture Sequence Memory (TBPSM) test for 8+ (episodic memory), the Pattern Comparison Processing Speed (PCPS) test for 7+ (processing speed), the Flanker Inhibitory Control and Attention (TBFICA) test for 8+ (executive function), the Dimensional Change Card Sort (TBDCCS) for 8+ (executive function). The Cognition Total Composite Score (COGTC), the Cognition Fluid Composite Score (COGFC) reflecting capacity for new learning, and the Cognition Crystallized Composite Score (COGCC) reflecting past learning were computed. Age-uncorrected scores were used to preserve the sensitivity to differences in age. Scores were corrected for site differences using a linear regression with age and sex as covariates. Linear mixed-effects repeated measures model (Baayen et al., 2008) with age and sex as covariates were performed using the lme4 package in R (version 4.1.2) (Bates et al., 2015) on all seven measures to confirm the expected significant improvements in cognitive performance over time. (Steinberg, 2005). To calculate the rate of change across time points, scores from TP1 were subtracted from TP2 to create the deltaT1 difference map, and TP2 was subtracted from TP3 to create the deltaT2 difference map.

### Statistical tests

#### DNAm and GM

A repeated measures mixed effects model (Baayen et al., 2008) was used to test the relationships between each of the seven CpGs and each of the seven GM networks across all three time points using the lme4 package in R (version 4.1.2) (Bates et al., 2015). The subjects’ GM network loadings (representing the networks’ variation across subjects and across time) were the dependent variables, the DNAm measures of the seven CpGs were the independent variables, while sex and baseline age were covariates. Initially, a family variable was added as a random effect to account for the impact of siblings within the study, but then removed from the model because it did not account for a significant amount of variance. Similarly, the differing time intervals between subjects’ data collection was also included as a possible confounder and ruled out. The results were Bonferroni corrected to control for Type I error at 5% for 49 tests (Emerging Infectious Diseases, 2015).

To further explore the multivariate relationships between the rates of change within the DNAm and GM networks, a MANCOVA analysis was performed using the jmv package in R (version 4.1.2) (R: MANCOVA) on data from deltaT1 and deltaT2 separately. The subjects’ loadings from the seven GM networks of the difference maps were the dependent variables, and the DNAm changes in seven CpG sites were the independent variables, with sex and baseline age as the covariates. Significant associations between GM networks and CpG sites were further tested individually for potential interactions with sex using the emmeans package in R (version 4.1.2) (RDocumentation).

#### DNAm and cognitive tests

Similarly, a repeated measures mixed effects model was used to test the relationships between each of the seven CpGs and each of the cognitive tests across all time points. In this model, the score from each cognitive test was the dependent variable and the DNAm of each CpG was the independent variable, while sex and baseline age were covariates. To further explore the multivariate relationships between the rate and direction of change in the DNAm and cognitive measures, a MANCOVA analysis was also performed on the changes in seven cognitive scores (dependent variables) and DNAm changes in seven CpG sites (independent variables) using data from deltaT1 and deltaT2 separately, with sex and baseline age as covariates.

#### GM and cognitive tests

In the same way a repeated measures mixed effects model was used to test the relationships between each of the seven networks of GM volume (the independent variable) and each of the cognitive tests across (the dependent variable) across all three time points, while sex and baseline age were covariates. A MANCOVA analysis was performed on the changes of cognitive scores (dependent variables) and GM networks (independent variables) using data from deltaT1 and deltaT2 separately.

## Results

### DNAm generally decreased over time

To determine which CpGs were experiencing significant change over time, a paired *t*-test was performed. The results showed that the DNAm of six of the seven CpG sites, located on *SLC12A9, NFASC, GRIN2D, STXBP5, GABRB3,* and *KCNC1,* all decreased in methylation across the three time points. The CpG site located on *CHD5* was the only CpG that increased in methylation significantly. All seven CpGs were located in active or highly active regulatory regions of their genes, indicating a role in regulating gene expression (Moore et al., 2013). There were no significant differences between the changes in methylation over time for these CpGs related to sex. Figures 1B–H shows the changes of the DNAm of these seven genes over time. A noticeable bimodal distribution in the DNAm of *GABRB3*, *NFASC*, *GRIN2D*, *STXBP5*, and *KCNC1* is likely due to genetic regulation from a proximal single nucleotide polymorphism (SNP): SNP allele type modulates methylation value of CpG site (Dupont et al., 2009). See Table 2 for detailed results. With the exception of *CHD5*, DNAm decreased over time.

### Cognitive performance improved over time

**FIGURE 2:**
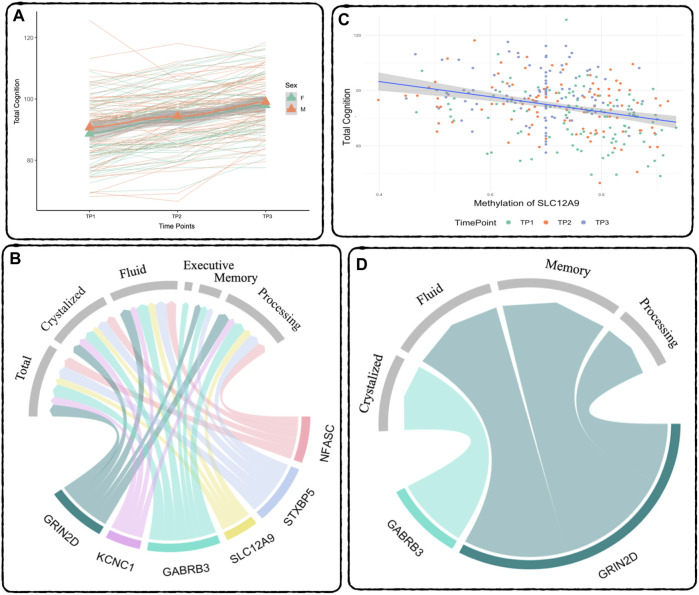
Relationship between DNAm and Cognition - **(A)** Cognition over time: Total Cognition (COGTC) across all three timepoints. The thick lines represent the mean for each sex, green is females, orange is males, the triangles are the mean at each timepoint. The thin lines represent each subject’s individual slope across time. Results of the repeated measures mixed effects model: between TP1 and TP2: t = 9.12, p < 2E-16, between TP2 and TP3: t = 18.80, p < 2E-16. **(B)** Repeated Measures Results: Summary of the significant relationships between DNAm and Cognitive Performance across all three time points. The width of the links corresponds to the effect size (t-statistic) of the relationship. **(C)** Demethylation of SLC12A9 Related to Increase in Total Cognition: The decrease in DNAm of SLC12A9 across all three time points was significantly related to the increase in total cognition (t = −3.76, *p* < 0.0002). **(D)** MANCOVA Results: Summary of the significant relationships between the rate of decrease in DNAm and the rate of improvement in cognitive performance. Cognitive measure labels: Total - COGTC, Crystalized - COGCC, Fluid - COGFC, Executive - TBDCCS, Memory - TBPSM, and Processing - PCPS. See Tables 4, 5 for complete results.

A repeated measures linear mixed effects analysis was used to verified the expected significant improvement across all cognitive measures (Bonferroni threshold: *p* < 0.007), although TBFICA and TBDCCS only showed significant improvement between TP2 and TP3. See Table 3 for a summary of the results. The following cognitive measures also showed significant baseline age effects: COGTC (t = 3.5, *p* < 0.0006), PCPS (t = 4.11, p < 7E-5), and TBDCCS (t = 3.48, *p* < 0.0007), where increased age is associated with increased cognitive performance. Figure 2A shows an example of the overall improvement seen in Total Cognition (COGTC) along the three timepoints.

**TABLE 3:** Cognitive Improvement over time: Summary of the significant results from the linear mixed effects models of cognition over time. Bonferroni corrected for multiple tests (uncorrected *p* < 0.001).

Cognitive test	T1 - T2, t-stat	T1 - T2, *p*-value	T2 - T3, t-stat	T2 - T3, *p*-value
COGTC	9.12	2E-16	18.8	2E-16
COGCC	6.19	2.3E-09	14.2	2E-16
COGFC	7.35	2E-12	14.4	2E-16
TBPSM	4.54	8.5E-06	6.85	5.3E-11
PCPS	6.34	1E-09	11.53	2E-16
TBFICA			3.6	4E-04
TBDCCS			4.40	1.6E-05

### Decreases in DNAm were associated with improved cognitive performance

A separate repeated measures linear mixed effect analysis showed that DNAm decreases in *SLC12A9, NFASC, GRIN2D, STXBP5, GABRB3,* and *KCNC1* were related to improvement in many of the cognitive measures, after Bonferroni correction for multiple testing. There were no significant relationships between the DNAm of *CHD5,* which was the only CpG to show an increase in methylation, and any of the cognitive measures. Figure 2B summarizes the significant linear relationships between DNAm and cognition. Figure 2C highlights the relationship between the decrease in DNAm of *SLC12A9* and the improvement in COGTC. The MANCOVA analysis revealed that the rate of DNAm decrease of *GABRB3* and was related to the improvements in COGCC during deltaT1. The rate of decrease in methylation of *GRIN2D* was associated with the rate of improvement in COGFC, TBPSM, and PCPS during deltaT2. There were main effects of sex (multivariate F = 4.63, *p* < 0.01) and baseline age (multivariate F = 4.73, *p* < 0.01) with regard to the rates of change in COGCC (univariate F = 6.94, *p* < 0.009 and F = 7.37, *p* < 0.008 respectively) in deltaT1. Females had a greater rate of improvement in their crystallized cognition composite score than males. Adolescents who were older at the initial assessment had a greater rate of improvement in their crystalized cognition composite score than those who were younger. Figure 2D summarizes the significant multivariate relationships between the rate of decrease in DNAm and the rate of improvement in cognition. See Tables 4, 5 for detailed results. Decreases in DNAm at six of the CpGs was significantly related to the improvements seen in total, crystalized, and fluid cognition as well as improvements in executive function, episodic memory, and processing speed.

**TABLE 4:** Complete Repeated Measures (RM) Results between DNAm and Cognition - The decrease in DNAm at the CpGs on these genes showed significant relationships with the improvement seen in the cognitive measures across all three timepoints. Threshold for Bonferroni corrections due to multiple testing was *p* < 0.001. Cognitive measure labels: Total - COGTC, Crystalized - COGCC, Fluid - COGFC, Executive - TBDCCS, Memory - TBPSM, and Processing - PCPS.

Genes	Cognitive test	RM t-stat	RM *p*-value
SLC12A9	Total	−3.76	0.0002
	Processing Speed	−4.09	6.2E-05
NFASC	Total	−5.56	7.8E-08
	Crystalized	−4.64	6.2E-06
	Fluid	−5.05	9.2E-07
	Processing Speed	−5.56	7.4E-08
GRIN2D	Total	−6.28	1.7E-09
	Crystalized	−5.23	5.1E-08
	Memory	−3.15	0.001
	Processing Speed	−5.87	1.4E-08
STXBP5	Total	−6.49	5.2E-10
	Crystalized	−5.49	1.1E-07
	Fluid	−5.77	2.7E-08
	Memory	−3.16	o.oo1
	Processing Speed	−6.04	6.1E-09
GABRB3	Total	−6.78	1.1E-10
	Crystalized	−5.64	5.8E-08
	Fluid	−6.09	5.4E-07
	Executive	−3.34	0.0009
	Memory	−3.48	0.0006
	Processing Speed	−6.34	1.9E-09
KCNC1	Total	−4.17	4.3E-05
	Crystalized	−4.35	2.3E-05
	Fluid	−3.38	0.0008
	Processing Speed	−3.39	0.0008

**TABLE 5:** Complete MANCOVA Results between DNAm and Cognition - The rate of decrease in DNAm at CpGs on these genes was related to the rate of improvement in cognitive performance in deltaT2. Multi/Univariate - Multivariate and univariate F-statistic and *p*-values listed. Cognitive measure labels: Crystalized - COGCC, Fluid - COGFC, Memory - TBPSM, and Processing - PCPS. Directionality of the relationships determined through linear regression.

Genes	Cognitive test	Multi/Univariate F-stat	Multi/Univariate *p*-value	Directionality of relationship
GRIN2D	Fluid	3.53/6.37	0.01/0.01	Negative
	Memory	3.53/7.18	0.01/0.009	Negative
	Processing Speed	3.53/3.75	0.01/0.05	Negative
GABRB3	Crystalized	3.23/4.58	0.04/0.03	Negative

### GM volume decreased across networks of parietal regions while the cerebellum and ventral pre-frontal cortex still show increased GM volume

**FIGURE 3:**
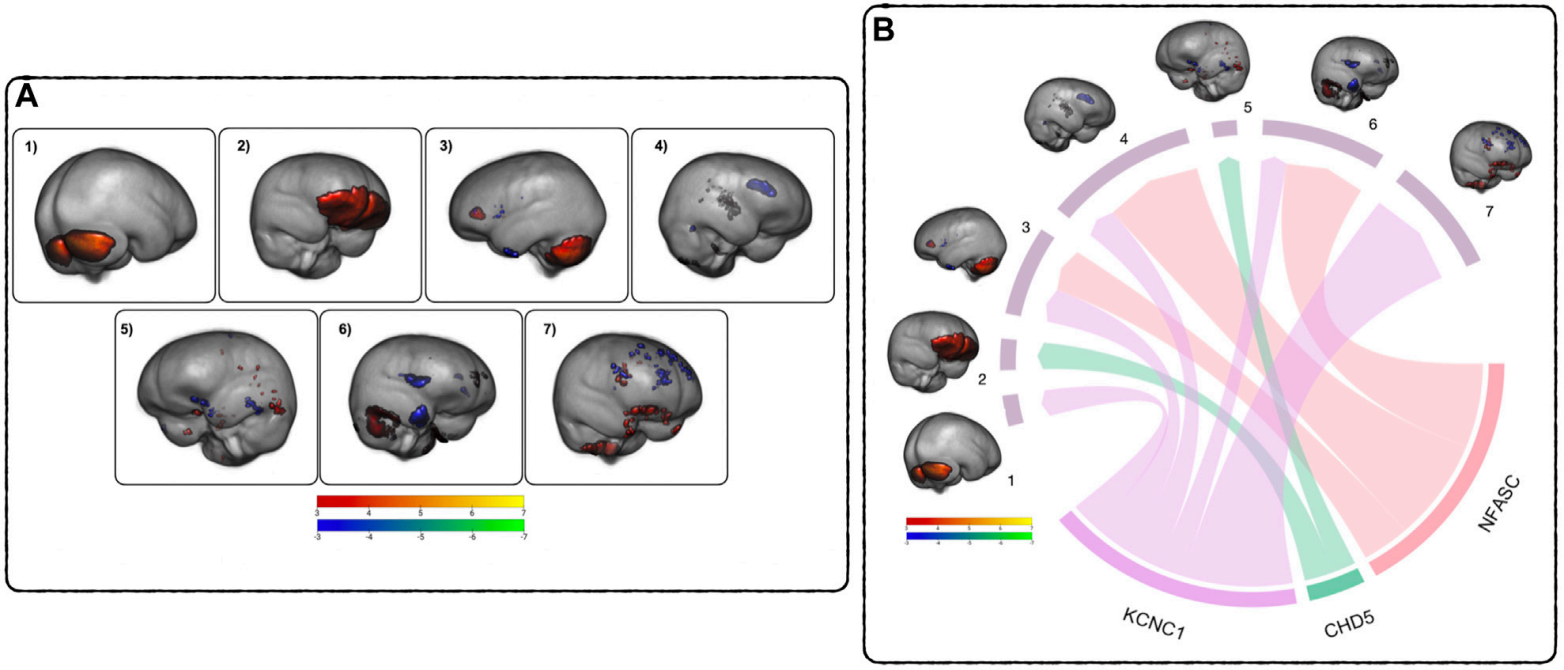
Relationship between DNAm and GM across time - **(A)** GM ICA results: Components 1–7, thresholded from −7 < z < −3 (blue to green) and from 3 < z < 7 (red to yellow), highlighting covarying differences in GM volume between TP1 and TP2. Blue - green are areas of GM volume decrease over time, red - yellow are areas of GM volume increase. See Supplementary Table S1 for a comprehensive list of regions. **(B)** MANCOVA Results Overview: Pictured are the significant relationships between the rates of change in DNAm and GM. The width of the links corresponds to the effect size (F-ratio) of the relationship. See Table 6 for complete results.

The ICA performed on the subject’s GM scans identified seven brain networks (referred to as components in Figure 3A) highlighting covarying regions of GM change within the subjects’ brains over time. These regions were identified using the Harvard-Oxford cortical and subcortical structural atlases (Frazier et al., 2005; Desikan et al., 2006; Makris et al., 2006; Goldstein et al., 2007) and the probabilistic cerebellar atlas (Diedrichsen et al., 2009). The first component (Comp1) highlights increases in cerebellar volume, including right and left crus I-II, left VI and VIIb, and vermiss VI, VIIIa-b, and vermiss crus II. The second (Comp2) highlights increases in the ventral prefrontal cortex. The third component (Comp3) consists of a network of increased GM volume in the cerebellum, thalamus, insula, and posterior cingulate gyrus covarying with decreases in GM volume in the anterior cingulate gyrus, lingual gyrus, and paracingulate gyrus The fourth component (Comp4) is a network of cerebellar increases covarying with decreases in the inferior frontal gyrus, the paracingulate gyrus, the temporal pole, the frontal pole, and left caudate**.** The fifth component (Comp5) comprises a network of increasing GM volume in the temporal occipital fusiform gyrus, the inferior temporal and middle temporal gyrus covarying with decreases in the posterior fusiform cortex, the lateral frontal orbital cortex, temporal pole, hippocampus, and middle frontal gyrus. The sixth component (Comp6) highlights a network of increasing cerebellar GM volume covarying with decreasing GM volume in the frontal operculum cortex and temporal pole. The seventh component (Comp7) consists of a network of increasing GM volume in the orbital frontal cortex and subcallosal cortex covarying with decreases in the superior frontal gyrus, the anterior cingulate gyrus, the frontal pole, the paracingulate gyrus, and the pars operculum of the inferior frontal gyrus. See Supplementary Table S1 for a detailed listing of the brain regions.

### The rate of DNAm decrease were associated with the rates of GM volume loss in several networks

To assess the relationships between the rates of change between DNAm and GM volume, a MANCOVA analysis of the respective difference maps was performed. The rate of decrease in DNAm of *NFASC* and *KCNC1* were related to the rate of decreases in the GM volume in three GM networks (Comp3, Comp4, and Comp6). Additionally, the rate of demethylation of *KCNC1* was related to decreases in Comp7. In contrast to this trend, the rate of increase in the DNAm of *CHD5* was related to the rate of decrease in GM volume in Comp2 and the rate of increase in GM volume in Comp5. Figure 3B summarizes the multivariate results between the rates of DNAm change and GM volume changes. The relationship between the rate of change between the DNAm of *KCNC1* and Comp1 had a significant interaction with sex (t = −4.768, *p* < 0.0001). In females, the rate of decrease of DNAm of *KCNC1* is related to the rate of increase in GM volume in Comp1. In males, the rate of decrease of DNAm of *KCNC1* is related to the rate of decrease in GM volume in Comp1. This interaction between sex and the rate of DNAm of *KCNC1* in the cerebellum is highlighted in Supplementary Figure S2. See Table 6 for detailed results. Decreases in the rates of demthylation of *NFASC* and *KCNC1* were associated with the rates of change in GM volume across varied regions of the brain, mostly associated with GM volume loss. The rate of increase of DNAm of *CHD5* was associated with the rate of change in GM networks that highlighted areas of GM volume increase.

**TABLE 6:** Complete MANCOVA Results between DNAm and GM volume - The rate of decrease in DNAm at CpGs on these genes was related to networks of GM change in the listed GM components. Multi/Univariate - Multivariate and univariate F-statistic and *p*-values listed. Directionality of the relationships determined through linear regression.

Genes	GM component	Multi/Univariate F-stat	Multi/Univariate *p*-value	Directionality of relationship
NFASC	Comp3	5.28/9.72	2.7E-05/0.002	Positive
	Comp4	5.28/21.35	2.7E-05/9.2E-6	Positive
	Comp6	5.28/17.61	2.7E-05/5.0E-5	Positive
KCNC1	Comp1	6.27/5.98	2.7E-6/0.02	Interaction
	Comp3	6.27/7.55	2.7E-6/0.007	Positive
	Comp4	6.27/7.08	2.7E-6/0.009	Positive
	Comp6	6.27/5.65	2.7E-6/0.02	Positive
	Comp7	6.27/22.21	2.7E-6/6.3E-6	Positive
CHD5	Comp2	2.31/6.01	0.03/0.02	Negative
	Comp5	2.31/4.64	0.03/0.03	Negative

### Decreases in DNAm and improvement in cognition weakly associated with GM volume decreases

To test the direct relationships between DNAm and GM volume over time, a repeated measures linear mixed-effects analysis was performed. The results did not survive correction for multiple testing, they did highlight a pattern of associations between networks of GM volume loss in Components 3, 4, and 6 with decreases in DNAm as well as improvements in cognition across time, which was very similar to the multivariate results between the rates of change in demethylation and networks of GM volume change. A summary of these results can be found in Supplementary Figure S1.

## Discussion

Investigation of the changes of DNAm in these seven genes and those relationships with GM volume and cognition across time in this adolescent cohort have given us a first look at some of the molecular underpinnings that may be partly responsible for driving the dynamic and profound changes associated with adolescence. The function and roles of these genes in the brain are highlighted in Table 7. The *post hoc* pathway analysis (see Supplementary Table S2) found significant pathways annotated to molecular-level functioning within the neuron, synapse, axon, and transporter complexes (Watanabe et al., 2017). Tissue enrichment from the FUMA analysis also confirmed elevated expression of these gene-enrichment pathways exclusively within the brain (Watanabe et al., 2017). More specifically, *SLC12A9*, *NFASC*, *GRIN2D*, *STXBP5*, and *CHD5* are all expressed throughout the brain, while *GABRB3* is expressed differentially in the following regions of the brain: cerebral cortex, hippocampus, cerebellum, thalamus, olivary body, and piriform cortex, and *KCNC1* is expressed in neurons that fire at high frequency, including neurons in the cerebellum, globus pallidus, subthalamic nucleus, substantia nigra, reticular thalamic nuclei, cortical and hippocampal interneurons (Uhlén et al., 2015). The location of these CpGs further support their role in regulating gene expression. The CpGs on *SLC12A9, NFASC,* and *GRIN2D* were located on the exons of the gene, which is associated with alternative splicing of the gene (Shayevitch et al., 2018). The CpGs of *CHD5, STXBP5, GABRB3,* and *KCNC1* were located on the introns of the genes, which is associated with changes in gene expression (Anastasiadi et al., 2018).

**TABLE 7:** Function and Role of the Genes - Listed are the function and role in the brain of the seven genes regulated by the seven CpGs selected in this study. Expression of these genes in the brain was cross-referenced using the Human Protein Atlas and verified through FUMA tissue expression analysis.

Genes	Function and role in the brain
SLC12A9	Solute carrier family 12 (potassium/chloride transporters), and specifically regulates the concentration of chloride in and around cells (including neurons) utilizing a potassium:chloride symporter (Gagnon and Delpire, 2013). The SLCA12 transporters are involved in cellular processes throughout the body, but in the brain have been functionally implicated in the signaling cascade of excitatory or inhibitory inputs (Smagin et al., 2021) and are associated with auditory processes, inhibition, locomotion, and peripheral nerve health (Gagnon and Delpire, 2013)
STXBP5	Encodes syntaxin binding protein 5, or tomosyn, a protein involved in the regulation of neurotransmitter release by stimulating the SNARE complex formation (Williams et al., 2011)
GABRB3	Encodes gamma-aminobutyric acid (GABA) receptor subunit beta-3, one of three subunits in the GABAA receptor, a ligand-gated ion channel for GABA, a major inhibitory neurotransmitter in the nervous system (PubChem, 2022a). GABAergic neurons are diverse and can be found throughout the brain, accounting for up to 25% of the neuronal composition of some regions (Schmidt-Wilcke et al., 2018). The role GABA plays in adolescence has been well-researched in non-human primates and rats, highlighting a critical role in the development of the prefrontal cortex through the fine-tuning of the excitatory/inhibitory balance (Caballero and Tseng., 2016). Non-human primates experience an adolescence-related maturation of GABAergic functionality marked by a shift in the composition and expression of the subunits of the GABAA receptors (Caballero and Tseng., 2016)
GRIN2D	Glutamate ionotropic receptor 2D, is one of four N-methyl-D-apartate receptor 2 (NMDAR2) subunits (PubChem, 2022b), and has been found throughout the brain playing key roles in synaptic plasticity that are essential to memory and learning (PubChem, 2022b). Research in rats has shown that while expression of most NMDA receptor sub-types increases over the course of maturity, NMDAR2D expression declines and becomes localized to the hippocampus and basal ganglia, while increasing in the cerebellum (Wenzel et al., 1996)
KCNC1	Encodes a potassium voltage-gated channel subfamily C member 1, which mediates the excitability of neuronal cells by regulating the influx of potassium (PubChem, 2022c). There is no current research on the role *KCNC1* plays in neural development or cognition, although mutations and loss of function are connected to intellectual disability and epilepsy (Kessi et al., 2020)
NFASC	Encodes neurofascin, an L1 family immunoglobulin cell adhesion molecule (L1CAM) (PubChem, 2022d). There are many isoforms of *NFASC* that are developmentally and spatially differentiated throughout the brain (PubChem, 2022d). Isoforms of *NFASC* have been found to be responsible for neurite outgrowth, organization of the axon initial segment and nodes of Ranvier, and neurite myelination (PubChem, 2022d). Recent animal research revealed that neurofascin regulates myelin targeting and sheath growth throughout the central nervous system (Suzuki et al., 2017)
CHD5	Encodes a neuron-specific protein of the chromodomain helicase DNA-binding protein family (National library of Medicine, 2023). These chromatin remodelers regulate DNA accessibility by altering nucleosomal structure, usually during neurogenesis, changing the expression of genes required in neuronal differentiation (Alendar and Berns, 2021). *CHD5* is involved in the regulation of neuronal genes linked to terminal neuronal differentiation, synaptic connectivity, and synaptic strength (Egan et al., 2013). The majority of research into *CHD5* is focused on early fetal brain development and its association with neoblast tumors (Alendar and Berns, 2021), little is known about its behavior during adolescence or its relationship with cognition

In our study, six genes, *SLC12A9, NFASC, GRIN2D, STXBP5, GABRB3,* and *KCNC1,* experienced a sharp decrease in methylation between TP2 and TP3. This dramatic rate of change was not matched by the changes seen in GM volume or cognitive performance, which showed more consistent decreases and increases respectively over time. To rule out that this difference in the rates of changes was caused by a batch effect within the methylation data, subsamples of subjects with methylation data of all three timepoints within the same batch were analyzed. The same precipitous drop in methylation between the last two timepoints was observed. This suggests a biological mechanism, maybe related to puberty status, worthy of future study.

The demethylation of these six genes which all play, to some degree or another, a role in the regulation of the excitatory and inhibitory signaling of the brain (Debanne and Poo, 2010; Arain et al., 2013; Morris, 2013; Smagin et al., 2021), were significantly related to the adolescents’ improvement in general, fluid, and crystalized cognition, as well as processing speed. Additionally, the demethylation of *GRIN2D*, *GABRB3*, and *STXBP5,* which are more directly involved in neurotransmission, was related to improvements of episodic memory over time. Current research shows that maturation of the GABAergic network plays a role in the restructuring of the hippocampus during adolescence (Caballero and Tseng, 2016). The demethylation of *GABRB3* was also related to improvement in executive function, consistent with research showing increased maturation of GABAergic networks is linked to improvements in executive function (Caballero and Tseng, 2016).

The CpG site on *CHD5* is the only one with increased methylation over time. The rate of change in *CHD5* was related to the rates of GM volume increases in the prefrontal cortex, and in a network of the temporal regions, as well as the rate of improvement in executive function. These influences could be due to the role this gene plays in the development of neural plasticity (Arain et al., 2013).

The networks of GM volume change from this cohort were consistent with existing research. At this stage of adolescence, the cerebellum and prefrontal cortex are typically increasing in volume (Tiemeier et al., 2010) while frontal and occipital poles, as well as dorsal parietal cortices, begin showing the maturation-related GM volume loss (Gogtay et al., 2004). The demethylation of *NFASC*, *KCNC1, GRIN2D*, *GABRB3*, and *STXBP5* across time were related, although not strongly (not surviving multiple comparison corrections) to the patterns in GM networks in components 3, 4, and 6 (Supplementary Figure S1), highlighting cerebellar increases covarying with decreases across the temporal, occipital, and frontal poles. These promising relationships coupled with the significant associations between the rates of DNAm and GM changes (Figure 3, the MANCOVA analysis) suggest a concurrent rather than causal association, particularly in light of recent research suggesting that GM volume loss measured in healthy adolescents is actually cortical thinning due to an increase in axon myelination (Natu et al., 2019). This is also supported by the significant relationships seen between the rates of GM volume “loss” and the rate of change in the demethylation of *NFASC*, which regulates myelination (Klingseisen et al., 2019). The rate of change of DNAm of *KCNC1* was also related to rates of GM volume change in various brain networks, possibly related to the increased excitability of adolescent brains (Debanne and Poo, 2010; Zanini et al., 2016), but more research would be needed to confirm this. Networks of GM volume changes across time also had trending relationships with the cognitive changes in crystallized and fluid cognition, as well as episodic memory and attention/inhibitory control. These relationships could also be due to the coincidental association of GM volume “loss” during adolescence happening in parallel to the changes in cognition, rather than driving them.

In addition to the strengths of this exploratory longitudinal study, there were some limitations. There was no measure for the stage of puberty for any time points. Future studies of methylation, brain development, and cognition should include this measure to further refine the model and take important hormonal changes into account. Attrition of subjects by the third time point created an imbalance between the deltaT1 and deltaT2 analyses, as well as limiting the number of subjects with complete data. While still very informative despite this limitation, this study should be seen as a preliminary glance at the interaction of dynamic changes in methylation, brain development, and cognition in adolescence. Replicating these results with more subjects should be undertaken.

There is also a scarcity of research on the exact impact the increases and decreases of methylation at these CpG sites have on the expression of the genes they regulate. Increases in methylation generally decreases gene expression, and decreases in methylation generally increases gene expression (Dupont et al., 2009), but not universally. This study provides a spotlight for further research into these particular CpGs and their effects on gene regulation within the brain, particularly during adolescence. Future work will also include looking at this same cohort using an epigenome-wide analysis, as well as include white matter and resting-state fMRI measures to capture a more complete picture of the influence of methylation on adolescent brain development and cognition.

## Conclusion

This small sample of seven CpGs highlight the dynamics of methylation and how they are related to some of the large-scale changes occurring in adolescence, such as the fine-tuning of the excitatory/inhibitory balance through shifts in the receptor subunits of *GRIN2D* and *GABRB3*, or the changes in synaptic strength and connectivity possibly driven by the changes in the regulation of *CHD5*, *STXBP5*, or *KCNC1*, or even the thinning of GM associated with changes in methylation of *NFASC*. The changes in cognition would also be supported by changes in synaptic connectivity, excitability, maturation of the excitatory and inhibitory networks, as well as the thickening of myelination that could be occurring in response to the changes in gene expression regulated by these CpGs. The findings of this study offer many new directions from which we can develop a more detailed understanding of the molecular underpinnings of these dynamics in the adolescent brain.

## Data Availability

The raw data supporting the conclusion of this article will be made available by the authors, without undue reservation.

## Supplementary material

SUPPLEMENTARY FIGURE S1 Repeated Measures Results: GM, DNAm, and Cognition - Pictured are the relationships between DNAm, GM volume, and Cognitive Performance across all three time points that trended toward significant, but did not survive Bonferroni correction (*p* < 0.001). The width of the links corresponds to the effect size (t-statistic) of the relationship. Components (Comp 3, 4, 5, 6, and 7 from bottom to top respectively) were thresholded from |z| > 3. Blue - green are areas of GM volume decrease over time, red - yellow are areas of GM volume increase. Cognitive measure labels: Crystalized - COGCC, Fluid - COGFC, Memory - TBPSM, and Attention - TBFICA.

SUPPLEMENTARY FIGURE S2 Interaction plot between methylation of KCNC1 and cerebellar volume - In females, the rate of decrease seen in methylation of KCNC1 is associated with the rate of increase in GM volume, as opposed to males, where the rate of decrease in methylation over time is associated with rate of decrease in GM volume. This divergence in the relationship between changes in methylation and changes in GM volume by sex was significant (t-ratio = −4.768, *p* < 0.0001). See Table 6 for complete results.

SUPPLEMENTARY TABLE S1 Comprehensive list of GM ICA results - Brain regions results of the ICA highlighting covarying regions of GM change within the subjects’ brains over time. These regions were identified by the MNI coordinates and the Harvard-Oxford cortical and subcortical structural atlases as well as the probabilistic cerebellar atlas.

SUPPLEMENTARY TABLE S2 FUMA Results - details of the enrichment analysis conducted using FUMA.
