# Supplementary material for: Epigenetic associations with adolescent grey matter maturation and cognitive development
Source: Front Genet. 2023 Jul 17;14:1222619. doi: 10.3389/fgene.2023.1222619 (PMC10390095; doi:10.3389/fgene.2023.1222619)
Supplement: Supplementary file 1 [file Table1.DOCX]

Supplementary Table 1

| Comp1 |  |  |  |
| --- | --- | --- | --- |
| Area | volume (cc) | Max Values | MNI (x, y, z) |
| Right Crus II | 23.5 | 7.2 | (16, -84, -38) |
| Left Crus II | 10.9 | 6.6 | (-17, -83, -40) |
| Left VIIb | 3.2 | 5.3 | (-22, -73, -46) |
| Left Crus I | 3.8 | 4.7 | (-16, -77, -31) |
| RIght Crus I | 0.5 | 3.9 | (29, -63, -34) |
| Left VI | 0.3 | 3.6 | (-28, -60, -32) |
| Vermis VI | 0.1 | 3.6 | (-2, -73, -28) |
| Vermis VI | 0.2 | 3.5 | (2, -73, -26) |
| Vermis VIIb | 0.1 | 3.5 | (-2, -69, -31) |
| Vermis VIIIa | 0.1 | 3.2 | (0, -69, -40) |
| Vermis Crus II | 0.1 | 3.1 | (2, -76, -33) |
| Vermis VIIIb | 0.1 | 3.0 | (0, -65, -41) |
| Vermis VIIIa | 0.1 | 2.9 | (1, -73, -38) |
| Lateral Occiptal Cortex, inferior division | 0.1 | -3.0 | (52, -70, -19) |
| Brain Stem | 0.1 | -3.0 | (-5, -39, -27) |
| RIght Crus I | 0.1 | -3.0 | (50, -46, -38) |
| Left I-IV | 0.1 | -3.1 | (-1, -44, -25) |
| Right Crus II | 0.4 | -4.0 | (46, -49, -46) |
| Comp2 |  |  |  |
| Frontal Orbital Cortex | 11.9 | 5.4 | (-26, 34, -18) |
| Frontal Pole | 20.4 | 5.4 | (27, 38, -17) |
| Frontal Medial Cortex | 11.2 | 5.4 | (3, 52, -19) |
| Subcollosal Cortex | 0.6 | 3.9 | (-3, 24, -13) |
| Paracingulate Gyrus | 1.8 | 3.8 | (-3, 37, -10) |
| Comp3 |  |  |  |
| Left VIIb | 8.4 | 6.3 | (-35, -68, -53) |
| Left Crus II | 4.8 | 5.7 | (-36, -77, -45) |
| Right VIIIa | 2.9 | 4.1 | (10, -66, -52) |
| Left Crus I | 2.2 | 4.1 | (-34, -81, -34) |
| Paracingulate Gyrus | 1.7 | 3.6 | (-2, 49, -4) |
| Cingulate Gyrus, anterior division | 0.9 | 3.5 | (2, 40, -2) |
| Inferior Frontal Gyrus, pars opercularis | 0.8 | -3.3 | (-56, 20, -3) |
| Temporal Pole | 0.5 | -3.9 | (-36, 5, -46) |
| Frontal Pole | 0.3 | -3.2 | (-26, 47, 4) |
| Caudate, left | 0.2 | 3.0 | (-5, 14, -1) |
| Subcallosal Cortex | 0.1 | 3.1 | (-1, 13, -2) |
| Temporal Fusiform Cortex, anterior division | 0.1 | -3.2 | (-35, 0, -50) |
| Postcentral Gyrus | 0.1 | -3.1 | (-63, -6, 20) |
| Right VI | 0.1 | -3.0 | (21, -63, -31) |
| Comp4 |  |  |  |
| Cingulate Gyrus, anterior division | 4.5 | -3.5 | (2, 18, 33) |
| Thalamus, left | 2 | 3.7 | (-18, -25, 15) |
| Cingulate Gyrus, posterior division | 1.4 | 3.3 | (-15, -40, 35) |
| Thalamus, right | 1.1 | 3.2 | (16, -19, 15) |
| Right IX | 0.4 | 3.9 | (5, -42, -37) |
| Insular Cortex | 0.4 | 3.4 | (-29, -29, 19) |
| Lingual Gyrus | 0.4 | -3.1 | (14, -65, -15) |
| Supramarginal Gyrus, posterior division | 0.3 | 3.1 | (42, -41, 12) |
| Left IX | 0.2 | 3.2 | (-6, -45, -38) |
| Superior Parietal Lobule | 0.2 | 3.2 | (29, -40, 41) |
| Caudate, right | 0.2 | 3.1 | (21, -28, 21) |
| Paracingulate Gyrus | 0.2 | -3.0 | (5, 28, 38) |
| Right VI | 0.2 | -3.1 | (17, -67, -17) |
| Precentral Gyrus | 0.1 | 3.1 | (-19, -33, 39) |
| Left I-IV | 0.1 | 3.0 | (-9, -38, -29) |
| Putamen, left | 0.1 | 2.9 | (-28, -14, 9) |
| Parietal Operculum Cortex | 0.1 | 2.9 | (45, -25, 28) |
| Frontal Pole | 0.1 | 2.9 | (31, 66, 0) |
| Comp5 |  |  |  |
| Temporal Occipital Fusiform Cortex | 1.7 | 3.6 | (39, -52, -12) |
| Temporal Fusiform Cortex, posterior division | 1.0 | -3.7 | (-42, -30, -18) |
| Frontal Orbital Cortex | 0.8 | -3.4 | (-16, 25, -18) |
| Inferior Temporal Gyrus, posterior division | 0.6 | 3.5 | (-60, -44, -22) |
| Middle Temporal Gyrus, posterior division | 0.6 | 3.2 | (-61, -29, -14) |
| Temporal Pole | 0.6 | -3.4 | (59, 8, -24) |
| Inferior Temporal Gyrus, temporooccipital part | 0.5 | 3.5 | (-55, -56, -24) |
| Planum Temporal | 0.4 | 3.3 | (-58, -21, 8) |
| Supramarginal Gyrus, anterior division | 0.3 | 3.2 | (-57, -25, 22) |
| Middle Frontal Gyrus | 0.3 | -3.0 | (-43, 5, 58) |
| Middle Temporal Gyrus, temporooccipital part | 0.2 | 3.3 | (41, -56, 13) |
| Precentral Gyrus | 0.2 | 3.2 | (-55, -3, 13) |
| Lingual Gyrus | 0.2 | 3.1 | (20, -56, -6) |
| Right VIIIa | 0.2 | 3.1 | (31, -37, -44) |
| Hippocampus, left | 0.2 | -3.2 | (-37, -27, -11) |
| Temporal Occipital Fusiform Cortex | 0.2 | -3.2 | (-30, -54, -16) |
| Superior Parietal Lobule | 0.2 | -3.1 | (-24, -43, 69) |
| Putamen, right | 0.1 | 3.2 | (14, 4, -16) |
| Temporal Fusiform Cortex, posterior division | 0.1 | 3.2 | (35, -7, -32) |
| Middle Frontal Gyrus | 0.1 | 3.2 | (-25, -4, 48) |
| Frontal Orbital Cortex | 0.1 | 3.0 | (22, 19, -18) |
| Lateral Occipital Cortex, superior division | 0.1 | 3.0 | (23, -64, 64) |
| Frontal Pole | 0.1 | -3.0 | (42, 37, 8) |
| Lateral Occipital Cortex, superior division | 0.1 | -3.1 | (27, -78, 16) |
| Cingulate Gyrus, anterior division | 0.1 | -3.0 | (9, -9, 46) |
| Comp6 |  |  |  |
| Frontal Operculum Cortex | 3.8 | -3.9 | (41, 12, 7) |
| Temporal Pole | 3.3 | -4.0 | (42, 7, -40) |
| Right Crus II | 1.7 | 4.8 | (47, -44, -44) |
| Right Crus I | 1.6 | 4.5 | (53, -47, -34) |
| Inferior Temporal Gyrus, posterior division | 1.3 | 4.5 | (56, -16, -36) |
| Left VI | 0.8 | 3.7 | (-41, -36, -35) |
| Parahippocampal Gyrus, anterior division | 0.8 | -3.7 | (25, 5, -35) |
| Left VIIb | 0.6 | 3.7 | (-40, -43, -52) |
| Frontal Pole | 0.4 | 3.2 | (-8, 70, 14) |
| Insular Cortex | 0.4 | -3.3 | (-33, 20, 7) |
| Middle Frontal Gyrus | 0.4 | -3.7 | (-39, 14, 28) |
| Right Crus VI | 0.3 | 3.4 | (28, -57, -36) |
| Inferior Temporal Gyrus, anterior division | 0.3 | -3.5 | (41, 0, -42) |
| Occipital Pole | 0.2 | 3.0 | (34, -96, -11) |
| Vermis VIIIb | 0.2 | -3.3 | (-6, -63, -41) |
| Middle Frontal Gyrus | 0.1 | 3.2 | (26, 16, 40) |
| Precentral Gyrus | 0.1 | 3.0 | (23, -24, 56) |
| Central Operculum Cortex | 0.1 | -3.4 | (44, 9, 7) |
| Vermis IX | 0.1 | -2.9 | (1, -60, -44) |
| Inferior Temporal Gyrus, temporooccipital part | 0.1 | -3.1 | (46, -43, -19) |
| Frontal Pole | 0.1 | -3.1 | (-38, 49, 0) |
| Parietal Operculum Cortex | 0.1 | -3.0 | (44, -32, 23) |
| Left IX | 0.1 | -3.0 | (-5, -63, -45) |
| Superior Frontal Gyrus | 0.1 | -3.0 | (-18, 16, 65) |
| Comp7 |  |  |  |
| Frontal Pole | 2.5 | -3.6 | (-26, 58, 26) |
| Subcallosal Cortex | 1.8 | 4.1 | (-3, 13, -18) |
| Paracingulate Gyrus | 1.8 | -3.3 | (2, 45, 24) |
| Inferior Frontal Gyrus, pars opercularis | 1.6 | -3.5 | (55, 15, 33) |
| Frontal Orbital Cortex | 1.5 | 3.7 | (20, 13, -18) |
| Inferior Frontal Gyrus, pars opercularis | 1.3 | 3.9 | (39, 9, 22) |
| Temporal Pole | 1.3 | 3.6 | (-39, 23, -35) |
| Right IX | 1.1 | 3.9 | (8, -59, -53) |
| Superior Frontal Gyrus | 1.0 | -3.5 | (20, 26, 60) |
| Cingulate Gyrus, anterior division | 1 | -3.3 | (-4, 40, 18) |
| Left IX | 0.9 | 4.1 | (-5, -58, -54) |
| Insular Cortex | 0.4 | 3.5 | (31, 7, 14) |
| Frontal Opercular Cortex | 0.3 | 3.7 | (33, 10, 16) |
| Inferior Temporal Gyrus, anterior division | 0.2 | 3.6 | (-52, 1, -36) |
| Paracingulate Gyrus | 0.2 | 3.0 | (15, 16, 42) |
| Temporal Fusiform Cortex, anterior division | 0.1 | 3.0 | (26, 2, -48) |
| Parahippocampal Gyrus, anterior division | 0.1 | 3.4 | (-14, 2, -23) |
| Caudate, right | 0.1 | 3.4 | (12, -2, 26) |
| Putamen, right | 0.1 | 3.1 | (27, 1, 12) |
| Subcallosal Cortex | 0.1 | 3.0 | (0, 12, -11) |
