# Supplementary figures and images for: Epigenetic associations with adolescent grey matter maturation and cognitive development
Source: Front Genet. 2023 Jul 17;14:1222619. doi: 10.3389/fgene.2023.1222619 (PMC10390095; doi:10.3389/fgene.2023.1222619)

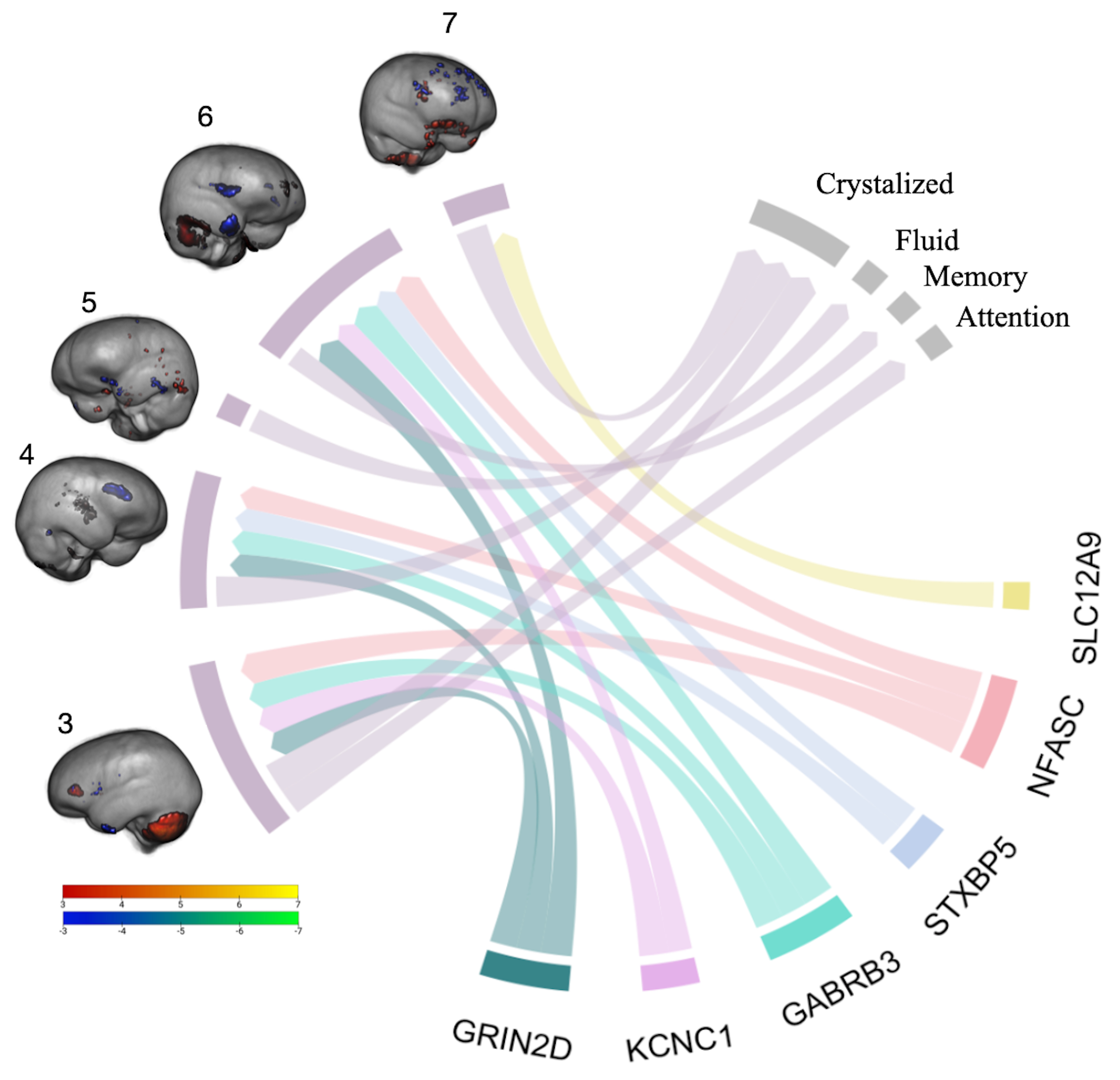

Supplement: Supplementary file 2 [file Image1.JPEG]

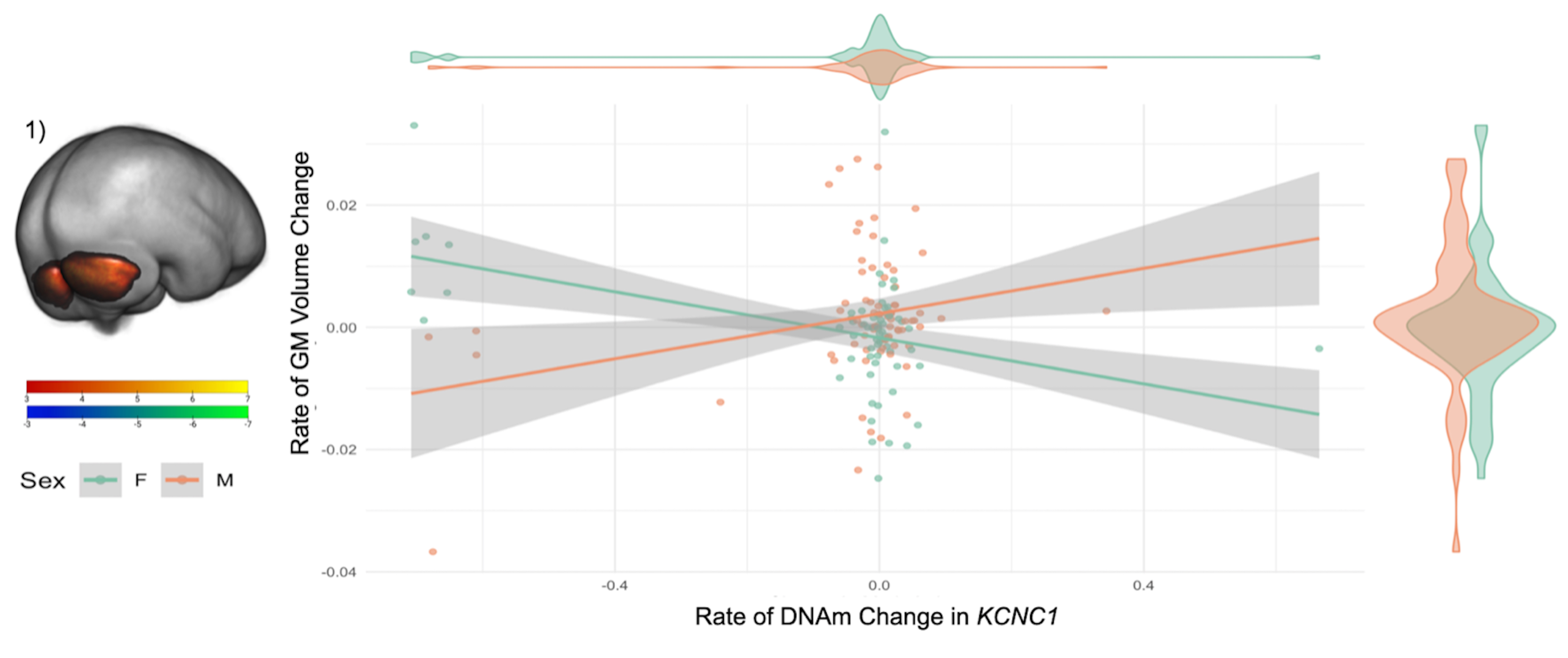

Supplement: Supplementary file 3 [file Image2.JPEG]
