# Supplementary material for: Epigenetic associations with adolescent grey matter maturation and cognitive development
Source: Front Genet. 2023 Jul 17;14:1222619. doi: 10.3389/fgene.2023.1222619 (PMC10390095; doi:10.3389/fgene.2023.1222619)
Supplement: Supplementary file 4 [file Table2.docx]

Supplementary Table 2:

| **Gene Set** | **N** | **n** | **P-adj** | **Genes** |
| --- | --- | --- | --- | --- |
| Transporter Complex | 334 | 3 | 0.01 | KCNC1, GABRB3, STXBP5 |
| Synaptic Membrane | 428 | 3 | 0.01 | KCNC1, GABRB3, STXBP5 |
| Main Axon | 68 | 2 | 0.01 | NFASC, KCNC1 |
| Intrinsic Component of Plasma Membrane | 1697 | 4 | 0.01 | NFASC, KCNC1, GABRB3, STXBP5 |
| Neuron Part | 1709 | 4 | 0.01 | NFASC, KCNC1, GABRB3, STXBP5 |
| Vesicle Membrane | 780 | 3 | 0.03 | NFASC, GABRB3, STXBP5 |
| Presynaptic Membrane | 161 | 2 | 0.04 | KCNC1, STXBP5 |
| Synapse Part | 932 | 3 | 0.04 | KCNC1, GABRB3, STXBP5 |
